# Altering Emulsion Stability with Heterogeneous Surface Wettability

**DOI:** 10.1038/srep26953

**Published:** 2016-06-03

**Authors:** Qiang Meng, Yali Zhang, Jiang Li, Rob G. H. Lammertink, Haosheng Chen, Peichun Amy Tsai

**Affiliations:** 1 Mechanical Engineering, University of Science and Technology Beijing, Beijing 100083, China; 2 Soft Matter, Fluidics and Interfaces, University of Twente, Enschede 7500 AE, The Netherlands; 3 State Key Laboratory of Tribology, Tsinghua University, Beijing 100084, China; 4 Department of Mechanical Engineering, University of Alberta, Edmonton, Alberta T6G 2G8, Canada

## Abstract

Emulsions–liquid droplets dispersed in another immiscible liquid–are widely used in a broad spectrum of applications, including food, personal care, agrochemical, and pharmaceutical products. Emulsions are also commonly present in natural crude oil, hampering the production and quality of petroleum fuels. The stability of emulsions plays a crucial role in their applications, but controlling the stability without external driving forces has been proven to be difficult. Here we show how heterogeneous surface wettability can alter the stability and dynamics of oil-in-water emulsions, generated by a co-flow microfluidic device. We designed a useful methodology that can modify a micro-capillary of desired heterogeneous wettability (e.g., alternating hydrophilic and hydrophobic regions) without changing the hydraulic diameter. We subsequently investigated the effects of flow rates and heterogeneous wettability on the emulsion morphology and motion. The experimental data revealed a universal critical timescale of advective emulsions, above which the microfluidic emulsions remain stable and intact, whereas below they become adhesive or inverse. A simple theoretical model based on a force balance can be used to explain this critical transition of emulsion dynamics, depending on the droplet size and the Capillary number–the ratio of viscous to surface effects. These results give insight into how to control the stability and dynamics of emulsions in microfluidics with flow velocity and different wettability.

Multiphase flow in porous media is a common theme and plays a significant role in soil remediation, enhanced oil recovery, membrane technology, and biological and medical applications. As a fundamental element of multiphase flow, emulsions—fine droplets of one liquid suspended in a different immiscible one—are widely used and studied^1^. For example, liquid-liquid encapsulates are fabricated and used for food, agrochemical, and pharmaceutical products^2^, oil recovery^3^, and drug delivery^4^,^5^,^6^. The stability of emulsions has a significant effect on these applications. However, precise control and manipulation of emulsion stability in microfluidics without an external driving force (e.g., acoustically or electrically) are rarely addressed. In this paper, we examine the effects of surface wettability on the stability of (oil-in-water) emulsions traveling in a micro-capillary (see Fig. 1), which mimics a simple pore-scale structure in a porous medium.

In contrast to polydisperse bulk emulsions produced by mechanical shear or agitation, stable monodisperse or double emulsions can be generated using microfluidics, including co-flowing^7^,^8^, T-junction, Y-junction^9^, or flow-focusing structure^10^,^11^, via shear flows between two immiscible phases^12^,^13^,^14^. The transport of emulsion slugs or droplets is of particular interest due to the promising applications of droplet-based microfluidics^8^,^9^,^12^,^15^,^16^,^17^,^18^,^19^,^20^,^21^,^22^. In a slug flow regime, the emulsion flows in the form of a liquid slug or long bubble, usually surrounded by a thin lubrication film of the continuous phase. This film thickness *h* (on the order of magnitude of microns) significantly depends on the Capillary number, *Ca*, a measure of the ratio of the viscous to capillary effect^23^,^24^,^25^,^26^. Conventionally, *Ca* = *μU/σ*, which is defined by the dynamic viscosity of the continuous phase, *μ*; the translational speed of the oil droplets, *U* (measured from the captured images); and the interfacial tension, σ, between the two phases. For instance, for water-in-oil droplets produced by a microfluidic flow-focusing structure, as *Ca* increases from 10^−4^ to 10, four different regimes of lubrication film can be delineated: wetting (*h* = *0*), thin film (*h* → 0), thick film (*h* ~ *Ca*^2/3^ for *Ca* > 10^−1^), and constant thicker film (*h/d* ~ 0.11)^27^. The majority of published studies on microfluidic emulsions are carried out using microchannels of homogeneous wettability. As revealed by complex contact line motions on flat or micro-structured open surfaces of heterogeneous partial wetting^28^,^29^,^30^, the heterogeneous wetting wall may exert a significant influence on droplet transport. However, the heterogeneous wettability effect on the emulsion morphology and stability is scarcely addressed in the literature^31^,^32^,^33^. In this paper, we elucidate the significant influence of heterogeneous surface wettability on the morphology and dynamics of microfluidic emulsions. Using experiments, a theoretical model, and simulations, we show that a critical convective time scale controls the emulsion with heterogeneous wettability stability at the micro scale.

## Experimental

**Figure 1:**
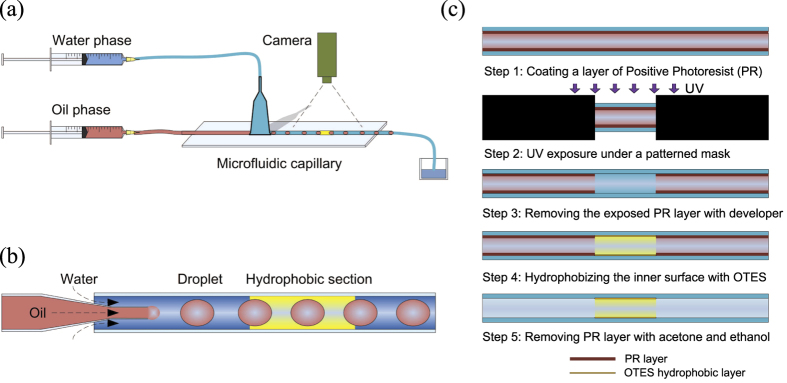
(**a**) Schematic diagram of the experimental setup of oil-in-water emulsions generated by a single co-flow microfluidic capillary, which has heterogeneous surface wettability. The continuous phase is 2% PVA aqueous solution, and the droplet is paraffin oil. (**b**) The glass micro-capillary, initially hydrophilic, is chemically modified to have a segment of a hydrophobic wall using a mono-layer of Octyltriethoxysilane (OTES) coating. (**c**) Outline of the experimental procedures to make a micro-capillary with a desired pattern of heterogeneous wettability.

We conducted the emulsion experiments using a single co-flowing microfluidic device, in the upstream section of a cylindrical glass capillary (see Fig. 1). In the micro-capillary, oil-in-water emulsions were generated by dispersion of an inner oil jet, injected through a concentrically-inserted, tapered micropipette puller, in a continuous aqueous phase. The inner liquid was paraffin oil while the outer liquid was an aqueous solution of 2 wt.% polyvinyl alcohol (PVA). The flow rates, *q*_*in*_ and *q*_*out*_, respectively, were controlled by two independent syringe pumps and varied to form oil droplets of different sizes. The interfacial tension between the two liquid phases was measured as σ = 15.2 ± 0.7 mN/m using the pendant drop method^34^. The dynamic viscosities of the oil and continuous aqueous phases were measured as *μ*_*o*_ = 21.5 ± 0.1 mPa·s and *μ* = 2.0 ± 0.1 mPa·s, respectively, using a Rotational Rheometer (Physica MCR 301, Anton Paar). We investigated the dynamics of the microfluidic emulsions using a microscope (magnification ≈10×) equipped with a high-speed camera.

Prior to the microfluidic experiments, we chemically modified a micro-capillary patterned with alternating hydrophilic and hydrophobic segments. The working concept combines a tailored-photolithography with self-assembled monolayer (SAM) chemistry. As a practical method of generating this pattern, we used positive photoresist (PR) to provide temporary protective layers to isolate the hydrophilic surfaces from the subsequent hydrophobization. Outlined in Fig. 1c are the essential procedures. First, we coated a dense PR layer on the inner wall of a hydrophilic micro-capillary. Second, we defined the boundary between the hydrophilic/hydrophobic segments by exposing the PR-coated micro-capillary to a parallel UV light under a photo-mask of the desired pattern. Third, the UV-exposed PR layer was washed away using a developer solution to expose the targeted hydrophobic segments. The remaining PR layer is dense and therefore resistant enough to isolate the covered surfaces from the latter SAM formation step. Fourth, we hydrophobized the exposed surface with an Octyltriethoxysilane (OTES) coating. Finally, we flowed a mixture of acetone and ethanol (1:1 v/v) in the micro-capillary to remove the silane residues and the coated PR layer, leaving the hydrophobic patterns intact. We verified the feasibility of this technique using a flat quartz slide, which is made from the same material and has the same properties as our capillary tubes. The static contact angles were measured as 27 ± 3° and 97 ± 5° with a water droplet of 2 *μ*L on the flat hydrophilic and hydrophobic surfaces, respectively. The corresponding contact angle hysteresis is about 70° (detailed materials and methods are described below).

## Results and Discussion

### Droplet morphology

**Figure 2:**
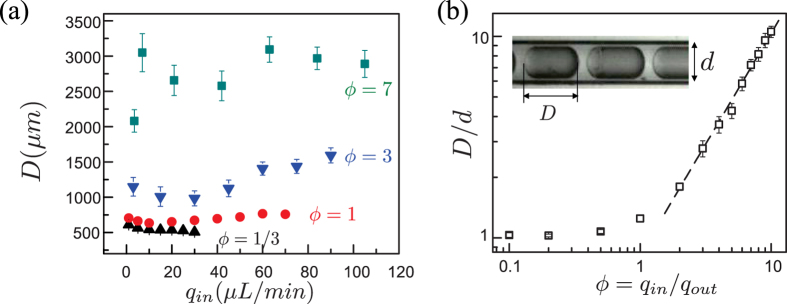
(**a**) Droplet length, *D*, as a function of the flow rate of inner (oil) phase, *q*_*in*_, for different flow ratios between the inner (oil) to outer (water) flow rate in an unmodified, hydrophilic capillary: *ϕ* = 1/3 (▲), *ϕ* = 1 (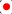), *ϕ* = 3 (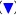), *ϕ* = 7 (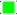). (**b**) Effect of flow ratio on the dimensionless droplet length *D*, normalized with the capillary diameter, *d*. The dashed line is the best fit of the power-law relation: *D/d* ~ *ϕ*^10/9^.

We first investigated the morphologies of oil-in-water droplets generated by single co-flow microfluidics in a glass capillary with homogeneous hydrophilic walls. Experimentally, we fixed the flow rate ratio, *ϕ* = *q*_*in*_/*q*_*out*_, between the inner oil phase (*q*_*in*_) and continuous aqueous phase (*q*_*out*_). We then recorded the droplet length *D*. Figure 2a shows the dependence of droplet length *D* on *ϕ*. Overall, *D* showed an increase with *ϕ*, ranging from 1/3 to 7. For each *ϕ*, *D* fluctuated around a nearly constant value, with a weak dependency on *q*_*in*_. Smaller *ϕ* gave rise to lower oscillation ranges because a large oil droplet (e.g., *ϕ* = 7) is more unstable due to a larger surface area and, hence, surface energy. Consistent with previous studies^35^, our experimental results also revealed that the droplet size exhibited a relatively weak dependency on *Ca*, for a fixed *ϕ*.

Control experiments were performed to validate the droplet length, *D*, as a function of *ϕ*. Here, *q*_*out*_ was fixed at 10 *μ*L/min, while the flow rate of the inner (oil) phase *q*_*in*_ was varied from 1 *μL/min* to 100 *μL/min*. Figure 2(b) shows the dependence of the dimensionless droplet size *D/d*, normalized by the capillary diameter *d* (580 *μ*m in our case), on the flow rate ratio, *ϕ*. For *ϕ* ≥ 2 the normalized droplet size *D/d* can be accurately described by a scaling relation dependent on *ϕ*: *D/d* ~ *ϕ*^1.10^, the best power-law fit in the range of *ϕ* between 2 and 10. Since *q*_*out*_ is fixed, *ϕ* ∝ *q*_*in*_ . *q*_*in*_ is proportional to the droplet volume *V*_*D*_, which can be estimated by *V*_*D*_ = *πd*^2^*D/*4 ~ *ϕ* because of the thin lubrication film. Consequently, *D* ~ *ϕ*, which is consistent with our experimental data, because the capillary diameter *d* is constant. The slightly higher power of 0.1 can be attributed to the thickness (or volume) of the lubrication film. In contrast, for a small *ϕ* range (between 0.1 and 1), the dependence of the dimensionless droplet length on *ϕ* is insignificant dependence, revealing a nearly constant of *D/d* ≈ 1.1. This limited drop size at small *ϕ* may be caused by the large tip diameter (202 *μ*m) of the co-flow device or the viscosity of the two phases preventing the formation of even smaller oil droplets less than 600 *μ*m. In summary, given a pair of fluids in a microfluidic co-flow device, the flow rate ratio, *ϕ*, and tip inner diameter, *d*, are the primary controlling parameters for generating emulsions of desired sizes.

### Emulsion dynamics

**Figure 3:**
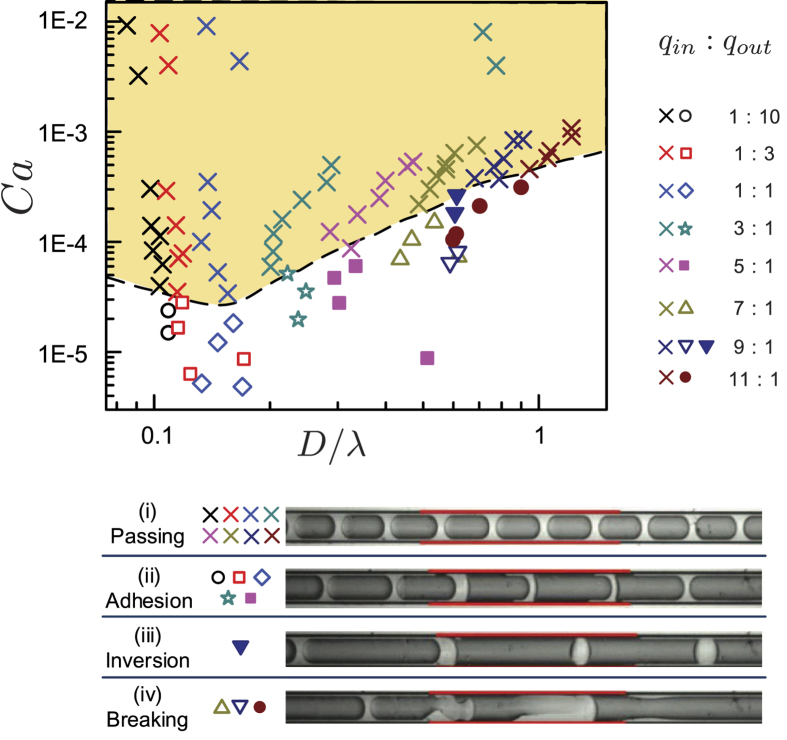
(Supporting movies) Phase diagram of (oil-in-water) emulsion dynamics after passing a hydrophobized section (marked in red along the micro-capillary), revealing the effects of droplet size and velocity, in terms of the Capillary number *Ca* = *μU/σ*, on the changing behaviors of the droplets. The upper (shaded) regime above the dashed line indicates (i) passing oil-in-water droplets without changing their size or speed (×) for different flow rates. In contrast, at lower speeds (below the dashed line) critical changes are observed, including (ii) adhesion of oil droplets on the hydrophobic wall with an increasing advancing contact angle (◦, ▫, ◊, 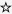, ■), (iii) inversion of oil droplets to become water-in-oil emulsions (▼), and (iv) break-up of the oil droplets with unstable lubrication films (Δ, ∇, ●). See Supplementary information for the details.

To investigate the influence of heterogeneous surface wettability, we systematically varied the flow rates of both phases while keeping *ϕ* constant. The heterogeneous wetting pattern used here is a hydrophobized segment of a length, *λ* (6 mm here) in the middle of a hydrophilic micro-capillary. Since the droplet size can be primarily controlled by *ϕ*, we varied the absolute flow rates, *q*_*in*_ and *q*_*out*_, while keeping the flow rate ratio *ϕ* unchanged. Using this method, we obtained droplets of different velocities, ranging from 3.6 × 10^−5^ to 7.0 × 10^−2^ m/s, while keeping the droplet size nearly constant. Figure 3 shows the phase diagram of the emulsion dynamics, in terms of *Ca* and *D/λ*, after passing the hydrophobic region from a hydrophilic wall.

The experimental results indicate that a critical change of emulsion stability occurs when the traveling speed of the oil droplets decreases below a critical speed for all *ϕ* in the explored range between 0.1 and 11. The results are reproducible using the same modified capillary tube. In other words, originally stable oil emulsions, surrounded by a thin water film along the hydrophilic wall, change their stability and dynamics at a low speed in the form of adhesion, inversion, or breaking. As shown in Fig. 3, these changing behaviors of oil droplets encompass (ii) adhesion of oil droplets on the hydrophobic wall with an increase in advancing contact angle, (iii) inversion of the droplets from the oil-in-water to water-in-oil form, and (iv) breaking of the oil droplets in the hydrophobic section. As illustrated in Fig. 3, these changing behaviors depend greatly on the speed and size of the oil droplets as they pass the hydrophobic segment. In comparison, a recent study showed that emulsions become inverted below a critical speed when traveling along an initially hydrophilic and subsequently hydrophobic capillary^33^. In that study, the effect of capillary diameter and droplet size on this critical *Ca* was investigated. Our experimental data, however, revealed more different outcomes in the emulsion dynamics. This difference may be caused by the use of different chemicals during hydrophobization and, hence, different dynamic contact angles and quantitative scaling relations.

As shown in the phase diagram of Fig. 3, for relatively small oil droplets produced with small *ϕ* ranging from 0.1 to 1, the oil-in-water emulsions are intact when traveling at a high speed, whereas they adhere on the hydrophobic wall at a lower rate or *Ca*. In this small-*ϕ* range, the droplet size of initial emulsions has an insignificant dependence on the droplet speed, as revealed by the first three nearly vertical data sets in Fig. 3. Initially, when oil droplets travel in the hydrophilic micro-capillary, the oil droplets do not touch the wall but are surrounded by a lubricating film of the aqueous solution. In this case, the dynamic contact angle of the wetting aqueous solution is 0°. A similar situation happens for the passing of unalterned oil droplets surrounded by a wetting film along the hydrophobic wall above a critical speed or *Ca*. For example, in the passing state (denoted by the symbols × in Fig. 3i), emulsions do not change the morphology in the hydrophobic segment of the micro-capillary; however, below a critical speed, the emulsions attain an adhesive state with a finite contact angle hysteresis, as shown in Fig. 3(ii). We measured the average advancing and receding angles of the adhesive oil-in-water emulsions along the hydrophobized segment (Fig. 3ii) to be 120 ± 6° and 47 ± 6°, respectively. This data was obtained from 17 different experiments with the flow ratio *ϕ* ranging from 0.1 and 5. In the adhesive state, the average contact angle hysteresis—the difference between the advancing and receding angles—is 73 ± 10°. This contact angle hysteresis of the traveling adhesive emulsions is consistent with the value of static contact angle hysteresis (70 ± 5°) measured with a water droplet on the flat hydrophilic and hydrophobic surfaces. For a relatively large droplet size, generated under more substantial *ϕ* between 3 and 11, the stable oil-in-water emulsions remain at a high droplet speed (or *Ca*). At low speed, these relatively large oil droplets invert, becoming water-in-oil emulsions or breaking up irreversibly. The inverted emulsions become water-in-oil (with a thin oil film presented on the hydrophobic wall) and hence has a dynamic contact angle of 180°. These phase changes–inversion and break-up pattern–destroy the original thin water film on the hydrophilic wall and result in a residual of oil film along the hydrophobic wall. The dashed line in Fig. 3 delineates the parameter regions of the unchanged and changing dynamics after emulsions pass the hydrophobic wall. In brief, fast-moving oil droplets are intact and unchanged when their parameters of *Ca* and *D* lie above this critical (dashed) boundary. In contrast, below this critical limit, oil-in-water emulsions alter their morphologies as the oil phase contacts the hydrophobic wall, resulting in adhesion, inversion, or break-up.

**Figure 4:**
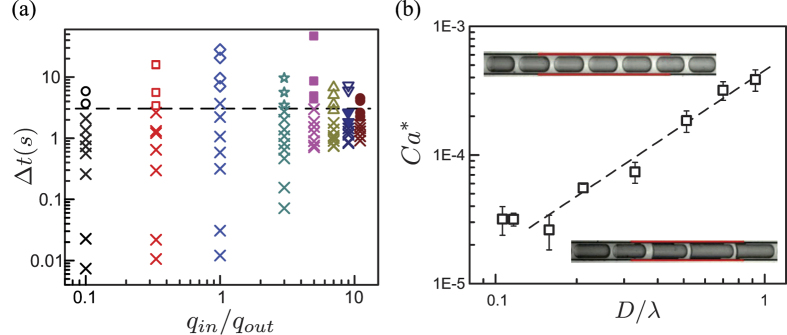
(**a**) Convective time scale, Δ*t* = *D/U*, of the oil-in-water emulsions (of speed *U* and length *D*) for different flow rates. The dashed line indicates the average critical convective time, Δt^*^, of the oil droplets. When Δ*t* < Δt^*^ the oil droplets are intact, passing without changing their dynamics, whereas for Δ*t* > Δt^*^, the droplets alter their morphologies with unstable lubrication film after traveling the hydrophobized section. (**b**) The critical capillary number for the oil droplets to cause the adhesion on the hydrophobic surface in the capillary. The solid line indicates a power-law fit of Ca^*^ ~ (*D/λ*)^*β*^, where *λ* is the length of the hydrophobized session, ≈10 *d*.

Remarkably, this critical parameter boundary reveals a universal, critical time scale, Δt^*^, for different flow rate ratios, *ϕ*. We define a characteristic timescale, Δ*t* = *D/U*, the droplet convective time scale. This characteristic, convective time scale also corresponds to the lifetime of the thin wetting film produced by the passage of the droplet in the initial hydrophilic channel. The same phase diagram as Fig. 3 is depicted in Fig. 4, but expressed in the phase space of convective time, Δ*t*, and flow rate ratio, *ϕ*. For each *ϕ*, there is a critical time scale Δt^*^, above which oil droplets change their morphologies, whereas below this time-scale, oil droplets remain intact. We experimentally found that this critical time scale is universal for the full range of *ϕ* explored: the average Δt^*^ = 3.0 ± 1.1 s. This critical timescale Δt^*^ can be associated with the typical dewetting timescale of the local thin wetting film (between the oil droplets and initial hydrophilic wall) due to the changes in wettability. The stability of the wetting film relies on its lifetime, i.e., convective time, which is shorter than the dewetting time scale, i.e., the critical Δt^*^. For instance, unstable thin films are obtained for large and slow-moving droplets, because the thin film lifetime is greater than its dewetting timescale, Δ*t* > Δt^*^, and thus is destabilized.

How can one explain the critical convective (or dewetting) time scale? Upon carefully examining our experimental movies, we noticed that the emulsion dynamics changed when the lubricating thin film was reduced in thickness to almost zero, i.e., when the oil droplet adhered to the hydrophobized section. Using highly magnified imaging, we measured the lubrication film (before the hydrophobized segment) for non-changing emulsion dynamics as *h* ≈ 6 *μ*m, ~*O*(10 *μ*m). This scale is larger than the interaction range of the van der Waals force, so the thin wetting film does not rupture. In contrast, when emulsion dynamics changes, the lubrication film is essentially very thin, approaching 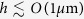 range, so the long-range attractive force between the oil phase and hydrophobized wall is increased, causing the thin film to rupture. Based on a finite element method using COMSOL, we also carried out numerical simulations that mimic our experiments. The computed lubrication films are consistent with our experimental results (see Materials and Methods). This consistence suggests that the critical time scale is associated with a wetting transition, i.e., destabilization, of the thin lubrication film.

The critical capillary number Ca^*^ is shown in Fig. 4b and is associated with the critical time scale and expressed in terms of droplet speed and size. Our experimental data show intact oil droplets at high speeds, *Ca* > Ca^*^, whereas morphology changes at low speeds, when *Ca* < Ca^*^. This critical flow velocity was found experimentally, clearly delineating the transition of unchanged to changed emulsion dynamics. For our experimental conditions of fluid properties and hydrophobic coating, this critical capillary number was experimentally found to be Ca^*^ = *α(D/λ)*^*β*^, with the best fit of the scaling power to be *β* = 1.40 ± 0.12 and prefactor *α* = 4.5 × 10^−4^ ± 7 × 10^−5^.

We further investigated a simplified theoretical model to explain this empirical power-law relationship. We estimated the critical condition for the thin film to rupture, by balancing the Laplace pressure and van der Waals forces acting on the slightly perturbed oil-water interface. The latter attractive force between the oil phase and hydrophobized wall can cause the water wetting film to rupture and is described by disjointing pressure, 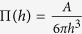, depending on the film thickness *h*, with the Hamaker constant *A*^36^,^37^,^38^. The Laplace pressure acting on a nearly cylindrical oil-water interface (of size *D*), but slightly perturbed with a modulation amplitude of the order of magnitude of the film thickness *h*, can be estimated by 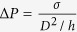. The thin lubrication film ruptures when 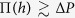, with a scaling relation of *D*^2^/*σ* ~ *h*^4^ at the transition point^33^. Here, instead of using the classical Taylor (*h* ~ *Ca*^1/2^) or Bretherton (*h* ~ *Ca*^2/3^) thin-film equations^23^,^39^, we use an empirical relation of the thin film thickness *h/d* ~ *Ca*^0.354^*We*^0.097^, recently obtained with high-resolution and non-intrusive measurements of thin-film thickness^40^. This scaling relationship has a slight Weber-number (*We*) weighting modification of the Bretherton description and gives a better approximation of various data than previous models, for wide ranges of Reynolds and Weber numbers^40^. Our experiments are in the ranges of *We* = 5 × 10^−8^–2 × 10^−1^ and *Re* = 10^−4^–2, calculated using the parameters of the oil droplet. We also performed numerical simulations of the thin film thickness by mimicking our experimental conditions of a microfluidic co-flowing structure. The simulations are based on a transient laminar two-phase flow (with the level-set model) using a finite element method. The detailed info and data can be found in the Supplementary Information. Using the empirical *We*-modified thin film thickness scaling, the film ruptures as the forces balance with the critical condition of Ca^*^ ~ *D*^1.41^, which agrees well with our experimental result, *β* = 1.40. However, still missing from the literature is a rigorous theory explicitly and quantitatively considering heterogeneous wettability as a function of the flow condition as well as the static, advancing, and receding contact angles.

## Conclusions

In summary, we demonstrated how to control the emulsion stability in microfluidics using heterogenous surface wettability. We also report the invention of an effective method to modify a micro-capillary with heterogeneous surface wettability of a desired pattern. For our parameter ranges, we found a universal critical convective time scale, Δt^*^, delineating the unaltered and changing emulsion dynamics. A fast-moving oil-in-water droplet, which has a thicker and hence more stable lubrication film, is stable without a change in emulsion dynamics when passing from hydrophilic to hydrophobic segments. In contrast, for a slow-traveling oil droplet, the thin-film is so thin and has an extensive lifetime and is thus unstable, causing the oil to adhere to the hydrophobic wall with adhesion, inversion, or break-up of the oil-in-water emulsions. This critical transition of the changing and unchanging emulsion dynamics is characterized by a critical Ca^*^, depending on the emulsion size and speed. A simplified model using a film thickness dependent force balance explains the dependence of this critical transition of emulsion stability due to the heterogeneous wetting surface, i.e., Ca^*^, on the emulsion size *D*. In brief, by tuning the droplet size and speed, as well as heterogeneous surface wettability, one can control the emulsion stability. Several useful applications can be extended from this work. For example, heterogeneous wettability is present in natural porous media and can now be fabricated in labs with the chemical modification methods reported. Another application is to address the problem of emulsion dynamics in porous media of heterogeneous wettability as emulsions can pass, adhere, inverse or break up at pore scale with wettability changes. A promising example is to control emulsion stability, such as de-emulsifying water-in-oil emulsions for oil production, using a surface-modified filtration system. Given the critical time-scale or Capillary number found and predicted here, one can tune the dynamics and stability of emulsions at the micro-scale. This critical control of emulsion stability is essential for and applicable to microfluidic, Lab-on-a-Chip, pharmaceutical, as well as oil and mining industrial applications.

## Methods

### Chemicals

Positive photoresist (PR1-4000A, Futurrex), Developer (RD6, Futurrex), Nitric acid (>65.0%, Sigma-Aldrich), n-Octyltriethoxysilane (OTES, 98%, Sigma-Aldrich), Hexane (>97.0%, Sinopharm Chemical Reagent Co., Ltd), Ethanol (>99.7%, Sinopharm Chemical Reagent Co., Ltd), and Acetone (>99.5%, Sinopharm Chemical Reagent Co., Ltd) were used as received for the fabrication of a wettability-patterned micro-capillary tube. Paraffin oil (Sinopharm Chemical Reagent Co., Ltd), Polyvinyl alcohol (PVA, Sigma-Aldrich), and Deionized (DI) water were used for the formation of the oil-in-water emulsions.

### Chemical modification of heterogeneous wettability

Figure 1c summarizes the key fabrication procedures of chemically modified micro-capillaries of heterogeneous surface wettability. The main criteria of successful alternating hydrophilic and hydrophobic regions are well-defined sharp boundaries, equivalent surface uniformity, and same capillary radius between the segments. As such, the influence on emulsion dynamics is only caused by the difference in surface energy, not due to the roughness or geometric changes. The essential method here is to use photoresist as a temporary protective layer to isolate the hydrophilic surfaces from the subsequent hydrophobization process.

As a target microfluidic capillary, we used a UV-transparent quartz tube (World Precision Instruments, Inc.) with an inner diameter of 580 *μ*m. The microtube was cleaned by a nitric acid flow at 15 *μ*L/min for 30 min. In Step 1, a PR (Positive photoresist) solution was pumped into the tubing at a flow rate of 1 *μ*L/min for 30 min to deposit a dense layer of PR along the inner channel wall. A stream of air flow was introduced at 5 *μ*L/min for 60 min to evaporate the solvents. The PR layer was subsequently solidified and strengthened in an oven at 70 °C for two days. In Step 2, the designed heterogeneous regions were patterned by placing the tubing horizontally under a glass mask for UV exposure (4–8 mW/cm^2^, China Electronics Technology Group Corporation) for 20 s. The length of the hydrophobic segments was 6 mm, designed by selectively patterning chromium on a glass mask. In Step 3, the developing solution was introduced into the tubing to dissolve the exposed PR layers (hydrophobic segment) and was sucked out by tissues. This step was repeated ten times to ensure that the exposed PR layers dissolved completely. The same procedure was followed by using distilled water to rinse away all the residues. The non-UV exposed PR layer remained after the developing step and was solidified in an oven at 70 °C for 30 min, creating an alternating PR layer covered tubing for hydrophobization.

In Step 4, an OTES/hexane (3 v/v %) solution was pumped into the tubing at 20 *μ*L/min for 30 min. The UV-exposed regions can be hydrophobized by OTES, and the PR layers can isolate these covered surfaces from OTES. In the last step, a hexane flow, followed by an acetone/ethanol (1:1 v/v) flow, was injected into the tubing to wash away all the unreacted OTES and deposited PR. The feasibility of this approach was successfully verified on a flat quartz plate first, to produce heterogeneous wettabilities. This method can be extended to make a micro-capillary with heterogeneous surface wettability of the desired pattern using suitable photo-masks, defining the hydrophilic and hydrophobic segments. Since the micro-capillary has azimuthal symmetry, the desired patterns would consist of alternating hydrophilic and hydrophobic patterns azimuthally along the tube.

## Additional Information

**How to cite this article**: Meng, Q. *et al.* Altering Emulsion Stability with Heterogeneous Surface Wettability. *Sci. Rep.* **6**, 26953; doi: 10.1038/srep26953 (2016).

## Supplementary Material

Supplementary Movie 1

Supplementary Movie 2

Supplementary Movie 3

Supplementary Movie 4

Supplementary Information
