# Supplementary material for: Altering Emulsion Stability with Heterogeneous Surface Wettability
Source: Sci Rep. 2016 Jun 3;6:26953. doi: 10.1038/srep26953 (PMC4891714; doi:10.1038/srep26953)
Supplement: Supplementary Information [file srep26953-s5.pdf]

Supplementary Information for Altering Emulsion Stability with Heterogeneous Surface Wettability

Qiang Meng[1], **Yali Zhang**[2], **Jiang Li**[1,§], **Rob G.H. Lammertink**[2], **Haosheng Chen**[3,†], **and Peichun Amy Tsai**[2,4,*]

[1]Mechanical Engineering, University of Science and Technology Beijing, Beijing 100083, China
[2]Soft Matter, Fluidics and Interfaces, University of Twente, Enschede 7500 AE, The Netherlands
[3]State Key Laboratory of Tribology, Tsinghua University, Beijing 100084, China
[4]Department of Mechanical Engineering, University of Alberta, Edmonton, Alberta, T6G 2G8 Canada

Appendix A: Thickness of thin lubrication films

Fig. S1 shows an estimation of the thickness of the lubricating thin film surrounding the oil droplets for our experimental conditions. The estimate uses the empirical relation of the thin film thickness $h/d \sim Ca^{0.354}We^{0.097}$ obtained with high-resolution and non-intrusive measurements of thin-film thickness.[1] Our numerical simulations using a finite element method (with COMSOL) are based on a transient laminar two-phase flow using the level-set model, with an inner oil fluid injected from a smaller capillary surrounded by water flow in a micro-capillary. The fluid density, dynamic viscosity, interfacial tension, and fluid domains are specified using our experimental conditions and measurements of the liquids used. The laminar two-phase level set method solves for the continuity and Navier-Stokes equations to conserve mass and momentum. Meanwhile, the interface position between the two fluids is tracked by solving a transport equation with the level set function,[2–4] with the surface tension force acting on the interface between the two fluids. We solved for incompressible Stokes flow, because of low-Reynolds number microfluidic flow, with a no-slip boundary condition between the fluids and the solid wall. The re-initialization parameter in the transport equation for tracking the interface was set to 0.07 m/s. The numerical results (shown in Fig. S1b and c) of film thickness are consistent with our few high-resolution experimental measurements of thin film and the estimation using the empirical results.[1]

Appendix B: Supplementary videos

Four representative movies of Fig. 3 (i-iv) are enclosed, showing different emulsion dynamics: (i) passing emulsions with unchanged dynamics, (ii) adhesive state, (iii) inversion, and (iv) breaking state with tunneling wetting film after the emulsions travel through the hydrophobic segment (marked in red in Fig. 3). The traveling speeds of the emulsions for Fig. 3 (i-iv) in the initial, hydrophilic micro-capillary are (i) 1.19 mm/s, (ii) 0.21 mm/s, (iii) 1.98 mm/s, and (iv) 0.78 mm/s, respectively.

References

1. Mac, M., Eain, G., Egan, V. & Punch, J. Film thickness measurements in liquid-liquid slug flow regimes. *Int. J. Heat Fluid Flow* **44**, 515–523 (2013).

2. Lafaurie, B., Nardone, C., Scardovelli, R., Zaleski, S. & Zanetti, G. Modelling merging and fragmentation in multiphase flows with surfer. *J. Comput. Phys.* **113**, 134–147 (1994).

3. Yue, P., Feng, J. J., Liu, C. & Shen, J. A diffuse-interface method for simulating two-phase flows of complex fluids. *J. Fluid Mech.* **515**, 293–317 (2004).

4. Olsson, E. & Kreiss, G. A conservative level set method for two phase flow. *J. Comput. Phys.* **210**, 225–246 (2005).

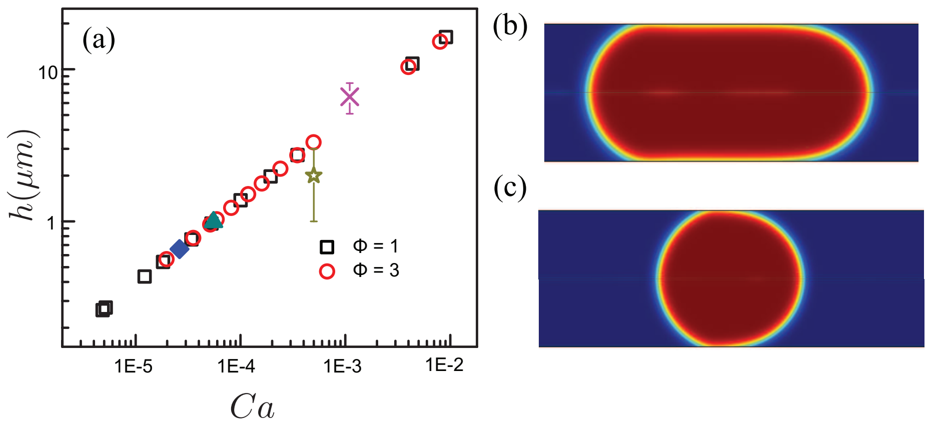

Figure S1. (a) The lubrication film thickness for our experimental results of $\phi = 1$ (□) and $\phi = 3$ (○), estimated using the empirical scaling relationship obtained by non-intrusive, high-resolution measurements: $h/d \sim Ca^{0.354}We^{0.097}$.[1] The symbols (♦) and (▲) indicate the film thickness at the critical Ca^*, below which emulsion dynamics are altered due to heterogeneous surface wettability for $\phi = 1$ and $\phi = 3$, respectively. Two representative simulation results, based on a laminar two-phase flow (level set model) of an oil droplet (in red) suspended in the water phase (in blue) at different Ca are shown: (b) one stable and thicker film, numerically estimated at $h = 6.6 \pm 1.5\mu$m at $Ca = 1.1 \times 10^{-3}$ and $\phi = 8.4$ (×), and (b) a thinner lubrication film of $h \approx 2 \pm 1$ μm at $Ca = 5 \times 10^{-4}$ and $\phi = 3.1$ ($\star$). The film thickness is measured using the narrowest distance between the oil (in red) and the wall. The transient color reveals a partial volume fraction of oil due to the level-set model and is indicated as the error for h.
